# m:Explorer: multinomial regression models reveal positive and negative regulators of longevity in yeast quiescence

**DOI:** 10.1186/gb-2012-13-6-r55

**Published:** 2012-06-21

**Authors:** Jüri Reimand, Anu Aun, Jaak Vilo, Juan M Vaquerizas, Juhan Sedman, Nicholas M Luscombe

**Affiliations:** 1 EMBL-European Bioinformatics Institute, Wellcome Trust Genome Campus, Cambridge CB10 1SD, UK; 2 University of Tartu, Institute of Computer Science, Liivi 2, Tartu 50409, Estonia; 3 Terrence Donnelly Centre for Cellular and Biomolecular Research, University of Toronto, 160 College Street, Toronto, Ontario, M5S 3E1, Canada; 4 University of Tartu, Institute of Molecular and Cell Biology, Riia 23, Tartu 51010, Estonia; 5 EMBL-Heidelberg Gene Expression Unit, Meyerhofstrasse 1, Heidelberg D-69117, Germany

## Abstract

We developed m:Explorer for identifying process-specific transcription factors (TFs) from multiple genome-wide sources, including transcriptome, DNA-binding and chromatin data. m:Explorer robustly outperforms similar techniques in finding cell cycle TFs in *Saccharomyces cerevisiae*. We predicted and experimentally tested regulators of quiescence (G_0_), a model of ageing, over a six-week time-course. We validated nine of top-12 predictions as novel G_0 _TFs, including Δ*mga2*, Δ*cst6*, Δ*bas1 *with higher viability and G_0_-essential TFs Tup1, Swi3. Pathway analysis associates longevity to reduced growth, reprogrammed metabolism and cell wall remodeling. m:Explorer (http://biit.cs.ut.ee/mexplorer/) is instrumental in interrogating eukaryotic regulatory systems using heterogeneous data.

## Background

Eukaryotic transcriptional regulation is a core cellular process that governs the expression of genes. Understanding gene expression is crucial in explaining complex biological processes including development, disease and cancer. Transcription factors (TF) are key proteins that activate or repress transcription by binding sequence-specifically to DNA in promoter regions of target genes. Mapping such regulatory networks and TF functions is therefore an important goal of current biomedical research. In complex vertebrate organisms like human, this task is hindered by enormous genomic space, numerous cell types, and distinct experimental procedures with data that is often unsuitable for direct comparison. The relatively simple unicellular model organism budding yeast (*S*. *cerevisiae*) serves as a platform for regulatory genomics. Multiple types of global-scale data of yeast gene regulation are available to date, including microarrays with TF deletion (ΔTF) strains [1,2], predictions of TF binding sites (TFBS) [3-5], and measurements of chromatin state such as nucleosome positioning [6]. These data appear to be complete, however the agreement between transcript expression and TF binding events remains modest [2,7]. While part of this controversy can be attributed to experimental and statistical noise, we may still lack significant details regarding the biological relationships among such heterogeneous information. Consequently high-throughput data constitute less reliable evidence and much functional knowledge is extracted from careful and expensive focused studies. Most TFs and their exact roles in cellular processes remain poorly understood. Therefore biologically meaningful computational analysis is an important challenge in deciphering cellular regulatory networks.

Computational prediction of TF function from gene expression and DNA binding data is an active area of research. Numerous algorithms have been published elsewhere, albeit few have been validated experimentally. Earliest approaches focused on a specific class of data and used alternative types of evidence for computational validation. For instance, microarray clustering followed by DNA motif discovery in gene promoters helped establish the genome-scale link between mRNA expression profiles and TF binding [8,9]. Similarly, analysis of cell cycle expression patterns of TF-bound genes led to recovery of cell cycle TFs [10]. More recent methods use statistical modeling to integrate multiple types of evidence. For example, ARACNE extracts transcriptional networks from numeric microarray data using mutual information [11], and MARINA is a down-stream method that identifies master regulators of these networks through association tests with TF binding target genes [12]. The SAMBA biclustering algorithm studies matrices of regulators and target genes, and highlights regulatory relationships between genes and TFs that co-occur in clusters [13]. The linear regression method REDUCE integrates numeric microarray data, DNA sequence and TF affinity matrices by modeling the linear relationship between gene expression levels and TF-DNA interactions [14]. The GeneClass algorithm additionally integrates information about gene function, as it constructs decision trees of discrete microarray profiles and TF binding sites to select predictors of process-specific genes [15]. While this method provides direct modeling of gene function, TFs and gene expression data are studied as independent predictors. Notably, none of the above methods take advantage of recent ΔTF microarrays that reveal regulator target genes [1,2]. Nested effects models are designed to extract regulatory networks from perturbation data [16], although integration of TFBS and gene annotations is not supported. Nucleosome positioning measurements also remain unexplored in all above approaches. In summary, additional computational efforts are required for meaningful integration of versatile biological data.

Here we propose a method m:Explorer that uses multinomial logistic regression models to predict process-specific transcription factors. We aim to provide the following improvements in comparison to earlier methods. First, our method allows simultaneous analysis of four classes of data: (i) gene expression data, including perturbation screens, (ii) TF binding sites, (iii) chromatin state in gene promoters, and (iv) functional gene classification. The model is based on the assumption that TF target genes from perturbation screens and TF binding assays are equally informative about TF process specificity. Second, we reduce noise by including only high-confidence regulatory relationships, and do not assume linear relationships between regulators and target genes. Third, we integrate detailed information to better reflect underlying biology: multiple subprocesses may be studied in a single model, and chromatin state data are incorporated into TF binding site analysis. TF target genes with simultaneous evidence from gene expression and TFBS data are highlighted separately. Fourth, our analysis is robust to highly redundant biological networks, as statistical independence is not required. We use univariate models to study all TFs independently and avoid over-fitting that is characteristic to many model-based approaches. This is statistically valid under the assumption that a complex model may be understood by examining its components.

To test our method, we compiled a comprehensive dataset covering most TFs of the budding yeast. We benchmarked m:Explorer in a well-studied biological system and establish its improved performance in comparison to several similar methods. Then we used the tool to discover regulators of quiescence (G_0_, stationary phase), a cellular resting state that serves as a model of chronological ageing. Experimental validations of our predictions revealed nine TFs with significant impact on G_0 _viability. Besides demonstrating the applicability of our computational method, these findings are of great potential interest to yeast biologists and researchers of G_0_-related processes like ageing, development and cancer.

## Results

### m:Explorer - multinomial logistic regression for inferring process-specific gene regulation

**Figure 1:**
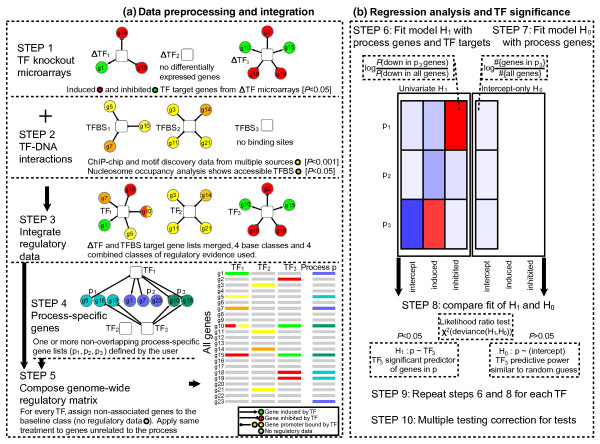
**Method summary**. **Figure 1A**: Data preprocessing. High-confidence TF target genes of four primary classes are collected from several datasets (Steps 1-2) and merged into composite lists with four extra classes for multiple lines of evidence (Step 3). Process-specific gene lists (Step 4) and TF target genes are assembled into a regulatory matrix (Step 5) such that unrelated genes are assigned to an additional "baseline" class. **Figure 1B**: TF significance tests with multinomial logistic regression models. For a given TF, the alternative model *H*_1 _is a univariate multinomial regression model that associates response (process genes) and one predictor (TF target genes), such that TF targets are linearly associated to probabilities of process gene classes (Step 6). The null model *H*_0 _associates response (process genes) to their relative frequency in the dataset (Step 7). Log-likelihood ratio test measures if *H*_1 _provides a better fit to data than the simpler *H*_0 _model (Step 8). All TFs are subject to independent testing (Step 9) and subsequent multiple testing correction (Step 10). TF, transcription factor knockout strain; ChIP, chromatin immunoprecipitation; TF, transcription factor; TFBS, transcription factor binding site.

Here we tackle the problem of identifying transcription factors that regulate process-specific genes (Figure 1). Our model m:Explorer uses three types of independent regulatory information to characterize target genes of TFs: gene expression measurements from TF perturbation screens, TF binding sites in gene promoters and DNA nucleosome occupancy in binding sites. The fourth input is a list of process-specific genes for which potential transcriptional regulators are sought.

The first stage of our analysis involves data preprocessing and discretization in which high-confidence TF target genes are identified from multiple sources (Figure 1A). We assumed that genes responding to TF perturbation are likely targets of the regulator. We previously analyzed a large collection of ΔTF microarrays, extracted genes with significant up or down-regulation (moderated t-test, FDR *p *≤ 0.05), and assigned these to perturbed regulators (Step 1, methods described in [2]). We also followed the assumption that TF binding in promoters is likely to indicate regulation of downstream genes, and binding sites in low nucleosome occupancy regions are more likely targets of TFs. We collected TF-DNA interactions from multiple datasets and classified genes as TF-bound if at least one dataset showed significant binding in 600 bp promoters (Step 2). We further categorized our TFBS collection into nucleosome-depleted TFBS (onesided t-test, FDR *p *≤ 0.05) and sites with no nucleosome depletion. Next we integrated TF target genes into a genome-wide matrix, by assigning non-related genes to a baseline class and creating extra classes for genes with multiple evidence (Steps 3, 5).

Besides regulatory targets of transcription factors, our method requires a list of process-specific genes for which potential regulators are predicted. These may originate from literature, additional microarray datasets, pathway databases or biomedical ontologies. Several non-overlapping lists of genes may be provided to integrate further information about sub-process specificity, sample treatment or differential expression. These genes are organized similarly to TF targets (Steps 4, 5).

The second stage of our analysis involves multinomial regression analysis of process-specific genes and TF targets (Figure 1B). It is a generalization of linear regression that associates a multi-class categorical response (process-specific genes) with one or more predictors (TF target genes). Through the logistic transformation, each gene is assigned a log-odds probability of being process-specific given its relation to a particular TF, as

$$g\left(y_{i}\right)=\text{log}\frac{p_{i,c}}{p_{i,C}}=\beta_{0,c}+\overset{K}{\underset{k=1}{\sum }}\beta_{k,c}x_{i,k},$$

where *y_i _*is the process annotation of the *i*-th gene, and *p_i,c _*is the probability that gene *i *is part of sub-process *c*, given a linear combination of *K *types of evidence *x *∈ *X *regarding TF target genes. All probabilities are computed relative to the baseline genes denoted by class *C*. The TF relation to process genes is quantified through regression coefficients *β *such that positive coefficients reflect a higher probability of TF target genes involving in the given process. Coefficients *β *are sought iteratively in maximum likelihood estimation. Likelihood reflects the estimated probabilities of all *N *genes belonging to their actual class, and thus provides a measure for model evaluation:

$$L\left(\beta |Y\right)=\overset{N}{\underset{i=1}{\prod }}\overset{C-1}{\underset{c=1}{\prod }}{\left(\frac{p_{i,c}}{p_{i,C}}\right)}^{y_{i,c}}p_{i,C}^{y_{i,C}},$$

where *y_i,c _*= 1 if *y_i _*is of class *c *and 0 otherwise, and the probability of gene-class relationship is computed as

$$p_{i,c}=\frac{\text{exp}\left(\beta_{0,c}+\sum_{k=1}^{K}\beta_{k,c}x_{i,k}\right)}{1+\sum_{c=1}^{C-1}\text{exp}\left(\beta_{0,c}+\sum_{k=1}^{K}\beta_{k,c}x_{i,k}\right)}.$$

Maximising the log likelihood *l *leads to optimal regression coefficients $\hat{\beta }$ and the corresponding likelihood value $\hat{l}$:

$$\left(\hat{\beta },\hat{l}\right)=\underset{\beta }{\text{arg}{\text{max}}_{\beta }}\overset{N}{\underset{i=1}{\sum }}\overset{C-1}{\underset{c=1}{\sum }}\left(y_{i,c}\left(\beta_{0}+\overset{K}{\underset{k=1}{\sum }}\beta_{k,c}x_{i,k}\right)\right)-y_{i,C}\text{log}\left(1+\overset{C-1}{\underset{c=1}{\sum }}\text{exp}\left(\beta_{0,c}+\overset{K}{\underset{k=1}{\sum }}\beta_{k,c}x_{i,k}\right)\right).$$

Here we implemented a statistical test to assess the process specificity of a given TF by comparing two multinomial regression models. The null model H_0 _: *g*(*Y*) = *β*_0 _is an intercept-only model where process-specific genes are predicted solely based on their frequency in the full dataset (Step 7). The alternative model H_1 _: *g*(*Y*) = *β*_0 _+ *β_k_X_k _*is a univariate model in which TF targets are also considered as predictors of process genes (Step 6). We use the likelihood ratio (LR) test with the chi-square distribution to compare the likelihoods of the two models, and decide if adding TF information substantially improves fit to data given its additional complexity (Step 8), as

$$P\left(H_{0}\right)=P_{\chi_{2}}\left(-2\left(\hat{l}\left(H_{0}\right)-\hat{l}\left(H_{1}\right)\right),\nu_{1}-\nu_{0}\right),$$

where *ν *corresponds to degrees of freedom and reflects number of model parameters. To predict all regulators to a process of interest, we test all TFs independently, correct for multiple testing and find TFs with significant chi-square p-values (Benjamini-Yekutieli FDR, *p *≤ 0.05).

In summary, m:Explorer uses the multinomial regression framework to associate process genes with TF regulatory targets from TFBS maps, gene expression patterns and nucleosome positioning data. Our method finds candidate TFs whose targets are especially informative of process genes, and thus may regulate their expression.

### Yeast TF dataset with perturbation targets, DNA binding sites and nucleosome positioning

We used m:Explorer to study transcriptional regulation and TF function in yeast, as it has the widest collection of relevant genome-wide evidence. First we compiled a dataset of 285 regulators that contains carefully selected target genes for nearly all yeast TFs from microarrays, DNA-binding assays and nucleosome positioning measurements. Statistically significant target genes from regulator deletion experiments originate from our recent reanalysis [2] of an earlier study [1]. High-confidence TFBS targets were assembled from earlier chromatin immunoprecipitation (ChIP) assays by Harbison *et al*. [3], *in silico *TFBS predictions [4,17], and recent refinements with protein-binding microarrays by Zhu *et al*. [5]. The data were further processed with *in vivo *nucleosome positioning measurements [6] to distinguish binding sites where lower nucleosome occupancy reflects open chromatin structure.

**Figure 2:**
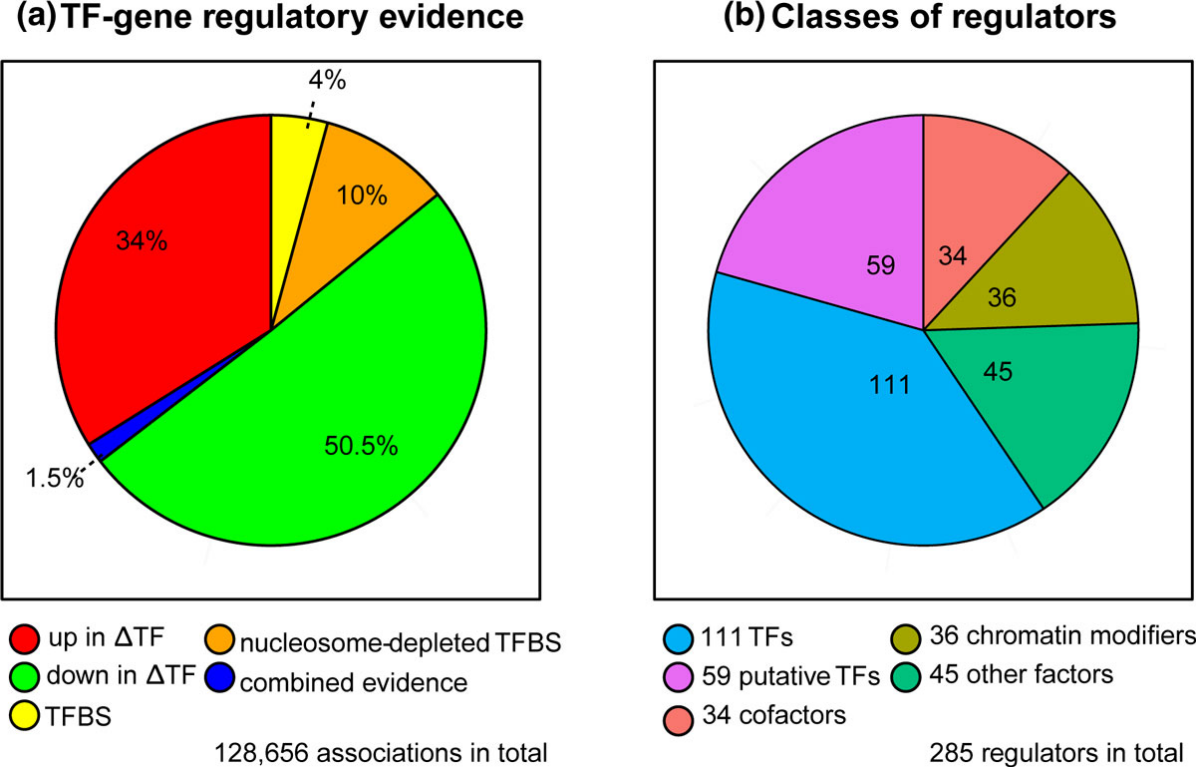
**Overview of yeast TF dataset**. **Figure 2A**: Distribution of TF target genes from gene expression data (red, green), TF binding data (yellow, orange), and combined evidence (blue). **Figure 2B**: Distribution of regulator classes in the TF dataset.TF, transcription factor knockout strain; TF, transcription factor; TFBS, transcription factor binding site.

Our dataset of 285 regulators contains 128,656 significant associations between regulators and target genes. Statistically reasoned cutoffs render our dataset sparse: it comprises high-confidence signals to 7.2% of approximately 1.8 million potential TF-gene pairs. The dataset includes 107 TF target sets with knockout data, 16 TFs with TFBS predictions and 162 TFs with both types of evidence. The majority of all gene-regulator associations (84%) originate from TF perturbation arrays (Figure 2A). As observed previously, the agreement between binding sites and ΔTF targets is low: only 1.5% of all high-confidence targets constitute both types of evidence. Along with 170 confirmed or putative DNA-binding TFs, our dataset covers cofactors, chromatin modifiers and other regulatory proteins (Figure 2B).

In conclusion, the yeast TF dataset is a useful resource for studying gene regulation.

### High-confidence recovery of cell cycle regulators

First we tested m:Explorer in a well-defined biological context. Cell cycle is a thoroughly described regulatory system with four consecutive phases: gap-1 (G1), synthesis (S), gap-2 (G2) and mitosis (M). Some of the earliest microarray experiments identified cell cycle-regulated yeast genes [18,19], and a computational analysis organized these into phase-specific groups [20]. Several focused studies have investigated the roles of individual cell cycle TFs [21-25], and a genome-wide experiment outlined the underlying regulatory network in its interconnected, circular nature [26]. Altogether, the core cell cycle network comprises nine transcriptional regulators (Swi4, Swi6, Mbp1, Ndd1, Fkh1, Fkh2, Swi5, Ace2, Mcm1, Additional file 1, Table s1).

**Figure 3:**
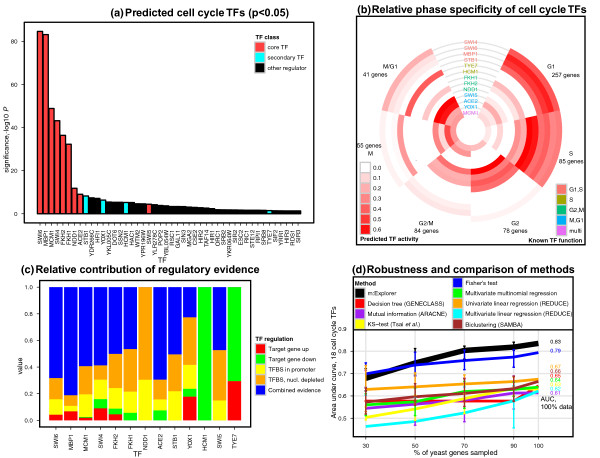
**Cell cycle TF prediction**. **Figure 3A**: Significance scores for 46 predicted TFs for cell cycle genes (FDR *p ***≤ **0.05). Red bars represent nine core cell cycle TFs and blue bars show secondary cell cycle TFs. **Figure 3B**: Predicted phase specificity for cell cycle TFs. Cell cycle phases are shown clockwise from top right, and TF activities across phases are shown in concentric circles (not drawn to scale). Intensity of red tone shows predicted proportion of TF activity from scaled regression coefficients. TF names are coloured according to their known phase of action, confirming the agreement between known and predicted functions of TFs. **Figure 3C**: Contribution of regulatory evidence in recovering 13 known cell cycle TFs. Bar color denotes class of evidence and bar height shows proportion of weight assigned to this class. **Figure 3D**: Performance and robustness comparison of m:Explorer and other methods. X-axis corresponds to proportion of genes presented to method, and Y-axis shows performance with the Area Under Curve statistic for 18 cell cycle TFs (AUC, 95% confidence intervals for 100 runs). m:Explorer values are plotted in wider, black lines. Numbers at the right end of curves reflect method performance with the full yeast dataset of 6253 genes. AUC, area under curve; G1, gap-1; G2, gap-2; M, mitosis; S, synthesis; TFs, transcription factors; TFBS, transcription factor binding site.

Here we applied m:Explorer and the TF dataset to select regulators to cell cycle genes. We focused on a recent tiling array study that measured genome-wide transcription during cell cycle at five minute resolution [27]. We used the list of 600 periodically expressed genes that contains specific groups for the four cell cycle phases and two checkpoints (G1, S, G2, G2/M, M, M/G1; 41-257 genes). This structured list of genes was then analyzed in a single m:Explorer run. We identified 46 statistically significant TFs (Benjamini-Yekutieli FDR *p *≤ 0.05, LR test from m:Explorer) including all nine core TFs (Figure 3A). Our results are ordered meaningfully, as eight of nine core TFs are ranked first (all *p *≤ 10^−9^). Besides core TFs, our results include at least four regulators that interact directly with the core TFs or act as secondary regulators. Notably, Stb1 forms a complex with G1/S TFs to affect gene expression in G1 [28], whereas Yox1 cooperates with Mcm1 to repress the expression of M/G1 specific genes [29]. The negative cell cycle regulator Ste12 is known to interact with Mcm1 in a specific pheromone-induced response [30]. In addition to cell cycle regulators, we found components of the transcriptional machinery, including the general transcription factor Taf14 and multiple subunits of the Mediator complex (Ssn2, Cse2, Srb2, Srb8, Gal11). Several chromatin modifiers are also present, e.g. the silent information regulators (Sir2, Sir3) carry out genome silencing and are related to replicative cell ageing [31]. We expected to see such regulators among our predictions, since their disruption is likely to affect any process that involves transcription.

Our method reveals additional details about cell cycle regulation. First, as we model all cell cycle phases in one run, relative TF phase activities can be quantified through regression coefficients (Figure 3B). For instance Swi4, Swi6 and Mbp1 make up the G1-S specific TF complexes MBF and SBF [21], and m:Explorer correctly highlights the phases with the strongest signal of regulatory activity. Second, we can assess the relative contribution of different kinds of regulatory evidence, and show that combined TFBS and ΔTF evidence are most informative of cell cycle regulation (Figure 3C). Third, simultaneous analysis of multiple sub-processes in a single multinomial model is advantageous to separate logistic models for each related subprocess, since the latter approach is more prone to false positive predictions (Additional file 1, Figure s1). We performed m:Explorer analysis for four cell cycle phases and two checkpoints separately and recovered all cell cycle TFs found by the multinomial model, however also retrieved a large number (28) of additional false positive TFs not associated to cell cycle. Despite the above, analysis of sub-processes showed that m:Explorer is applicable to relatively small gene lists, for instance Mcm1 and Yox1 are correctly recovered as regulators of M-phase through only 55 informative genes.

Next we compared m:Explorer with eight similar methods for predicting TF function in regulatory networks (Additional file 1). As no other method allows exact replication of m:Explorer models, we used combinations of discretized and numeric gene expression, TF binding and cell cycle data as required (Table 1). Method performance evaluation was carried out with the Area Under Curve (AUC) statistic that accounted for 18 cell cycle TFs (Additional File 1, Tables s1-s2). To measure performance robustness, we also conducted a benchmark in which random subsets of input data were presented to each method (30, 50, 70 and 90% of yeast genes, 100 subsets each). The simulation shows that m:Explorer substantially outperforms all tested methods in recovering cell cycle regulators (Figure 3D, AUC = 0.835 for 18 TFs, AUC = 0.996 for nine core TFs). Our method is reasonably accurate even when 50% of genes are discarded from the analysis (mean AUC = 0.747). The only method with comparable performance is the Fisher's exact test, a standard statistic for detecting significant biases in frequency tables. Comparison of m:Explorer and Fisher's test shows that our method is less prone to false positive discovery from randomly shuffled data (Additional File 1, Figure s2), and less dependent on microarray discretization parameters (Additional File 1, Figure s3). Fisher's test also prohibits the combined use of multiple features like gene expression, TF binding, nucleosome occupancy, and cell cycle phases. Simultaneous modeling of all data types in m:Explorer is likely to contribute to the demonstrated advantage over other approaches.

**Table 1:** Summary of comparison with similar methods

Method	Software	Reference	ΔTF expression	TFBS	Gene function	Nucleosomes
Univariate multinomial regression	m:Explorer		Discretized	Discretized	Discretized	Discretized
Multivariate multinomial regression			Discretized	Discretized	Discretized	Discretized
Decision tree	GeneClass	[15]	Discretized	Discretized	Discretized	
Kolmogorov Smirnov test		[10]		Discretized	Numeric^1^	
						
Mutual information	ARACNE	[11]	Numeric		Discretized^4^	
Fisher's exact test			Discretized^3^	Discretized^3^	Discretized	Discretized^3^
Biclustering	SAMBA	[13]	Discretized	Discretized	Discretized^4^	Discretized
Univariate linear regression	REDUCE	[14]		Numeric^2^	Numeric^1^	
						
Multivariate linear regression	REDUCE	[14]		Numeric^2^	Numeric^1^	

1. Gene expression values from cell cycle time-course. 2. Promoter sequence data and TF-DNA binding affinity matrices. 3. TF target genes treated as union of gene expression and TFBS targets. 4. Fisher's test used for finding cell cycle TFs in constructed network or clustering. TF, transcription factor knockout strain; TFBS, transcription factor binding site.

In conclusion, the cell cycle analysis showed that our approach successfully recovers a well-characterized regulatory system from multiple lines of high-throughput data. m:Explorer greatly outperformed several similar methods and showed robustness to incomplete data.

### Computational prediction of TFs for quiescence entry and maintenance

Next, we applied m:Explorer in a less familiar biological context to create experimentally verifiable hypotheses about TF function. We focused on the transcriptional mechanisms that govern cell quiescence (G_0_, reviewed by Gray [32] and Kaeberlein [33]). G_0 _is a cellular resting state with no proliferation, silenced genomes, reduced metabolism and translation, and greater stress resistance. Studying G_0 _has proven difficult and related regulatory programs remain elusive.

**Figure 4:**
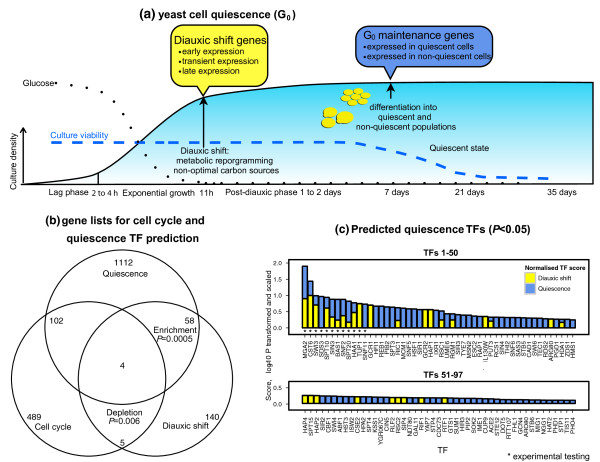
**Quiescence (G**_0_**) regulation and prediction of candidate TFs**. **Figure 4A**: G_0 _in *S. cerevisiae *occurs when a saturated culture is depleted of nutrients. As exponential growth stops with the exhaustion of glucose, cells pass diauxic shift and switch to slower respiratory growth. Growth is halted in post-diauxic phase, cells differentiate into quiescent and non-quiescent populations and enter G_0_. Culture viability starts decreasing rapidly as G_0 _progresses. Two sets of process-specific genes were used in independent m:Explorer runs: genes expressed during diauxic shift (yellow box), and genes expressed in G_0 _cultures (blue box, G_0 _maintenance). **Figure 4B**: Venn diagram for cell cycle genes, diauxic shift genes, and G_0 _maintenance genes. Statistically significant enrichment is observed between diauxic shift and quiescence genes (Fisher's exact test, *p *= 0.0005, 62 genes). **Figure 4C**: Log-scaled TF scores from diauxic shift and G_0 _maintenance predictions (top - TFs 1-50, bottom - TFs 51-97). Bar color represents fraction of final score attributed to G_0 _profile (diauxic shift - yellow; G_0 _maintenance - blue). First 12 TFs highlighted with asterisks were selected for experimental testing. TF, transcription factor.

Quiescence of yeast cells can be experimentally induced as a response to prolonged starvation (Figure 4A). When glucose is depleted in exponentially growing cultures, growth rate is reduced as cells pass diauxic shift in which metabolic reprogramming initiates respiration of non-optimal carbon sources. Nutrients are depleted in post-diauxic phase, resulting in halted growth and differentiation to quiescent and non-quiescent cell populations [34]. The quiescent fraction of homogeneous cells may survive for extended periods of time, while the ageing heterogeneous non-quiescent fraction dies on further starvation. Consequently, culture viability starts decreasing rapidly in later stages of G_0_. Induction and inhibition of quiescence has been associated to several highly conserved signalling pathways, including protein kinases A and C (PKA, PKC), TOR and Snf1 [32].

Here we studied two public microarray datasets and executed m:Explorer in two independent rounds. First, we retrieved 207 diauxic shift genes in three distinct subgroups of early, transient and late expression from the dataset by Radonjic *et al*. [35]. Second, we used 594 genes and 676 genes characteristic of quiescent and non-quiescent cells from the study by Aragon *et al*. [36] (Figure 4B). We identified 29 and 82 statistically significant candidate TFs in the two runs, log-transformed the scores and produced a final list of 97 G_0 _regulators (Figure 4C). A large number of regulators is expected, as G_0 _entry is thought to comprise large-scale cellular reprogramming [35]. Several top-ranking TFs have high scores in both m:Explorer predictions. This ranking is not an artifact of the overlap between diauxic shift and quiescence genes. Although the two lists comprise a considerable number of common genes (*n *= 62, *p *= 0.0005, Fisher's exact test), these were not sufficient for predicting a similar collection of G_0 _TFs, as m:Explorer analysis with the 62 genes only provided in a single significant TF (Mga2, *p *= 0.0005, LR test from m:Explorer).

In summary, the result of this analysis is an inclusive, prioritized list of candidate G_0 _TFs that serves as a resource for hypothesis generation and experimental testing.

### **Experimental validation reveals super-wildtype and essential G**_0 _**TFs**

**Figure 5:**
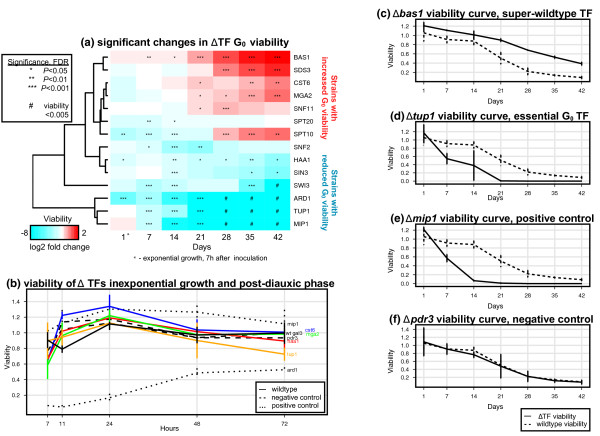
**Experimental validation of G**_0 _**TFs**. **Figure 5A**: Viability of ΔTF strains in contrast to wildtype and negative controls, shown as a hierarchically clustered heat-map. Strains are shown vertically and time-points in days horizontally, coloured cells denote log fold change of viability (red - increased viability, blue - decreased viability). Asterisks denote statistical significance of fold change (linear regression, LR test, FDR *p *≤ 0.05). Inviable strains with viability less than CFU = 0.005 are labeled with the symbol #. Time-point on day one corresponds to exponentially growing cells, measured seven hours after inoculation. **Figure 5B**: High-resolution viability curves of the first 72 hours of the time-course covering exponential growth, diauxic shift and post-diauxic phase. A subset of ΔTF strains (coloured solid lines), as well as wildtype strains (black solid lines) and controls (dashed and dotted lines) are shown. **Figure 5C**: G_0 _viability curve of super-wildtype strain Δ*bas1*. **Figure 5D**: Viability curve of G_0_-essential strain Δ *tup1*. **Figure 5E**: Viability curve of positive control strain Δ*mip1*. **Figure 5F**: Viability curve of negative control strain Δ*tup1*. TF, transcription factor knockout strain; FDR, false discovery rate.

Next we selected top 12 high-scoring TFs from our predictions for experimental testing. In total, 17 different strains were grown to G_0 _and assessed for viability in six consecutive weekly measurements (Figure 5A). We included deletion strains of candidate TFs (Δ*mga2*, Δ*cst6*, Δ*swi3*, Δ*sds3*, Δ*spt10*, Δ*sin3*, Δ*bas1*, Δ*snf2*, Δ*spt20*, Δ*haa1*, Δ*tup1*, Δ*snf11*), positive controls (Δ*ard1*, Δ*mip1*), negative controls (*Δpdr3*, Δ*gal3*) and wildtype strains (Additional file 2). The viability of some strains was additionally monitored in five measurements over the first 72 hours of growth (Figure 5B, Additional file 3). To confirm the timeframe of exponential growth and diauxic shift, we measured culture density and glucose levels of wildtype strains during 48 hours of growth (Additional file 1, Figure s4). To distinguish TFs with significant viability deviations, we used a linear error model that accounted for viability in wildtype and negative control strains as well as experimental batch effects.

All tested strains showed significant deviances from background viability at different stages of the quiescence time-course (Figure 5A). The deletion strains of Bas1, Sds3, cst6, Mga2, and Spt10 show consistently greater viability in G_0_, indicating that their normal presence in wildtype cells suppresses viability and hastens cell ageing (Figure 5C). We refer to these knockout phenotypes as super-wildtypes (WT+). In particular, Δ*bas1 *strains are on average 1.7-4.5 times more viable than wildtype in weeks 3-6 of quiescence (all FDR *p *≤ 10^−4^, LR test from error model). The transcription factor Bas1 is involved in the regulation of amino acid and nucleic acid metabolic pathways [37], and cst6 is related to chromosome stability and non-optimal carbon source regulation [38,39]. Spt10 and Sds3 are chromatin modifiers involved in genome silencing [40,41], and Mga2 regulates fatty acid metabolism, transcriptional silencing and response to low oxygen [42-44]. Deletion of Sds3 of the Sin3-Rpd3 histone deacetylase complex has been associated to increased chronological cell ageing [45].

The deletion strains Δ*tup1*, Δ*swi3*, Δ*haa1 *are significantly less viable than wildtype in quiescence (Figure 5A). In particular, Δ*tup1 *and Δ*swi3 *strains become inviable in later stages of G_0 _(viability ≤ 0.005) and can be considered essential for survival in this cell state (Figure 5D). Two further strains Δ*spt20 *and Δ*snf2 *are less viable in early quiescence, while Δ*sin3 *shows later deviations. With the exceptions of Sin3 and Haa1, corresponding null mutants are previously known for decreased or absent respiratory growth. Tup1 is a general inhibitor of transcription that establishes repressive chromatin structure [46]. Other factors are also involved in regulation of chromatin, transcription and genome stability, such as Swi3 and Snf2 of the SWI-SNF complex [47], Sin3 of Sin3-Rpd3 complex [48] and Spt20 of the SAGA complex [49]. While the factors have not been specifically described in the context of quiescence, disruption of their global functions is likely to affect this cellular state. Besides the above, the reduced G_0 _viability of Δ *haa1 *potentially relates to its role in regulating cell wall proteins [50].

Before entering quiescence, most tested TF strains have similar viability to wildtype strains, suggesting that their function in regulating viability is specific to G_0 _(Figure 5B). During exponential growth at seven hours after inoculation, only three strains including the positive control Δ *ard1 *are significantly less viable. Ard1 encodes an N-terminal acetyltransferase subunit that guides genome silencing, and Δ*ard1 *fails to enter G_0 _as observed previously [51]. In contrast, the other positive control Δ*mip1 *is as viable as wildtype in exponential phase, and more viable in post-diauxic phase. Mip1 encodes a mitochondrial DNA polymerase subunit required for cell respiration [52], and Δ*mip1 *loses viability in a similar manner to Δ *tup1 *(Figure 5E). Curiously, Δ*spt10 *is less viable in exponential growth phase and early quiescence, while its viability exceeds wildtype after week three of our time-course. The negative control strains Δ*gal3 *and Δ*pdr3 *expectedly show no major deviations from wildtype viability (Figure 5F). The TFs are related to alternative carbon metabolism and drug resistance, respectively [53,54], and show non-significant scores in m:Explorer predictions of G_0 _TFs. Finally, our glycerol growth assays confirm the respiratory properties of tested strains (Additional file 1, Table s3) and mostly agree with previous studies [55,56]. However, in contrast to those reports, our data indicate that Δ*cst6 *is viable on glycerol and indeed displays increased G_0 _viability.

According to our knowledge, most of our predicted TFs are not recognized as quiescence regulators. However previous functional evidence refers to processes important in quiescence, and hence lends confidence to our experimental observations. Besides uncovering novel regulators of viability in G_0_, our experiments show that m:Explorer provides biologically meaningful prediction of regulator function.

### **Functional enrichment analysis explains roles of G**_0 _**TFs**

To gain insight into G_0 _gene regulation of validated TFs, we performed a functional enrichment analysis of their G_0 _target genes. We focused on quiescence genes defined by Aragon *et al*. [36] and identified the subset of genes that were bound by at least one WT+ TF or showed differential gene expression in at least one WT+ ΔTF microarray [2]. Target genes were then scored by product of differential expression p-values across all WT+ ΔTF microarrays and ranked such that genes with most dramatic transcriptional changes were prioritized. The target gene list for viability-deficient TF strains was complied in a similar fashion. We expect that ΔTF differential expression is informative of regulatory relationships in quiescence. The strains underlying microarray profiling are genetically identical to the strains in our G_0 _experiments, although the former assays were performed with exponentially growing cells. Intersection of known quiescence genes with target genes of validated G_0 _TFs, and subsequent prioritization according to differential expression, is therefore likely to highlight high-confidence TF targets and functional relationships. To investigate this in detail, we then used the ordered gene list analysis of g:Profiler [57] to study the functional importance of significance-ranked target genes of WT+ and viability-deficient TFs.

**Figure 6:**
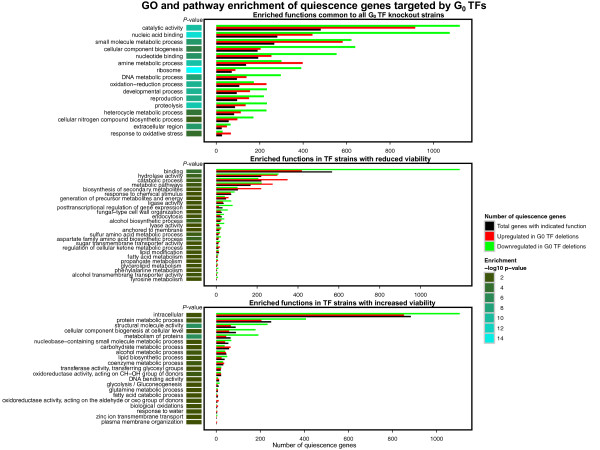
**Gene Ontology and pathway analysis of G**_0 _**TFs**.Quiescence genes with differential expression in G_0 _TF knockout microarrays were studied with ranked enrichment analysis in g:Profiler. Statistically significant non-redundant functional Gene Ontology categories are shown (hypergeometric test, FDR *p *≤ 0.05). Black bars correspond to total number of G_0 _genes in a given category, and coloured bars show the number of times these genes were up-regulated (red) or down-regulated (green) in related G_0 _ΔTFs, according to knockout data. Enrichment p-value is shown in vertical colour strips on the left. Top: functional enrichments of quiescence genes associated to all G_0 _TFs; middle: functional enrichments specific to TFs with reduced viability phenotype; bottom: Functional enrichments specific to WT+ TFs with increased viability phenotype. GO, Gene Ontology; TF, transcription factor.

Our analysis revealed 62 non-redundant Gene Ontology categories and KEGG and Reactome pathways with statistically significant enrichment in quiescence-related targets of G_0 _TFs (FDR *p *≤ 0.05, hypergeometric test, Figure 6). A number of functions were found to be enriched in TF targets corresponding to both viability phenotypes, suggesting that improved and reduced viability in quiescence may involve common regulatory pathways. The most significant results include the KEGG pathway of ribosome (*p *= 10^−15^), proteolysis (*p *= 10^−11^), reproduction (*p *= 10^−9^) and oxidation-reduction process (*p *= 10^−10^). Other functions are informative of TFs responsible for reduced G_0 _viability. For instance, metabolic and catabolic genes (*p *= 0.0070 and *p *= 0.0035) are mostly up-regulated, while genes related to cell wall organization are inhibited (*p *= 0.030). In contrast, WT+ TFs with increased G_0 _viability associate to down-regulation of protein metabolic genes (*p *= 10^−7^) and modulation of alternative energy pathways such as fatty acid catabolism (*p *= 0.034) and glutamine metabolism (*p *= 0.047).

Taken together, the above results associate to known mechanisms of quiescence and provide clues of the regulatory programs of predicted G_0 _TFs. Inhibition of growth through down-regulation of ribosome genes has been linked to increased replicative lifespan [58]. Efficient cell wall remodeling and response to increased oxidative stress are essential prerequisites of quiescence entry and survival [32]. Expectedly, increased viability appears to correlate with reduced metabolism, as related genes show opposite expression patterns in corresponding strains. Further discussion on G_0 _TFs and related pathways can be found below.

## Discussion

### **Function of G**_0 _**regulators**

It is tempting to speculate about the role of identified quiescence TFs in modulating quiescence signalling, as links between the factors and global G_0_-related pathways are apparent in our dataset. Our findings of WT+ regulators are especially intriguing, since their normal presence in wildtype cells reduces viability in quiescence and causes increased chronological ageing. From the perspective of evolutionary maintenance, WT+ regulators should engage in significant cellular functions that compensate for such negative properties.

As an example of G_0 _regulation, protein kinase A (PKA) mediates nutritional signals to the cell and is known as an inhibitor of quiescence [32]. Its primary regulatory subunit Bcy1 acts as an inhibitor of the pathway, and mutations in Bcy1 cause viability loss and death in G_0 _[59,60]. This double negative regulatory mechanism provides a potential explanation to observed viability phenotypes. In our TF dataset, Δ*mga2 *has significantly higher levels of Bcy1, potentially allowing more starving cells to pass into quiescence. The G_0_-essential Tup1 and Swi3 knockout strains have depleted levels of Bcy1 and as a possible consequence, we observe reduction and loss of viability. As another example, protein kinase C (PKC) guides cell wall remodeling in response to starvation and its activity is required for G_0 _entry [61]. The cell wall biosynthesis enzyme Gsc2 is a downstream target of PKC [62] and part of the gene expression signature of quiescent cells [36]. In ΔTF microarrays, Δ*mga2 *and Δ*cst6 *strains have elevated levels of Gsc2, while Δ*swi3 *and Δ*tup1 *show inhibition of PKC upstream of Gsc2 (Additional file 1, Figure s5). Other genes with known function in G_0 _appear to be regulated by WT+ and viability-deficient TFs. Notably, the conserved superoxide dismutase (SOD) genes are responsible for neutralizing oxidative damage of mitochondrial respiration. In yeast, SOD genes are required for G_0 _survival and extend chronological lifespan when over-expressed [63,64]. Induced levels of Sod2 expression in Δ*cst6 *may explain our observations of increased G_0 _viability.

Several confirmed G_0 _TFs are also associated to mammalian gene regulation.Cst6 carries the DNA-binding domain of CREB, an extensively studied TF that regulates a variety of processes, including cell survival and proliferation, cellular metabolism, and synaptic plasticity of long-term memory [65]. Bas1 is homologous to the MYB TF that regulates stem and progenitor cells and appears as an oncogene in multiple tumour types [66]. Chromatin modifier complexes Swi/Snf, Sin3/Rpd3 and SAGA are also broadly conserved, for instance Swi3 homolog SMARCC1 is involved in versatile functions, including neural stem cell renewal and differentiation [67]. As the yeast quiescence model associates to hallmark cancer properties of cell cycle control, proliferation and differentiation, further analysis of our Findings may reveal intriguing links to cancer biology.

### Applicability and validity of m:Explorer

Here we present the robust computational method m:Explorer for predicting functions of gene regulators from high-throughput data. We applied a model that probabilistically accounts for multiple types of regulatory signals and functional gene annotations. To take advantage of abundant genome-wide data and powerful experimental approaches, we present a case study for predicting transcription factors (TF) in the unicellular budding yeast. However, our method is not restricted to yeast and even not to these classes of data and regulators, being easily scalable to more complex regulatory systems of vertebrate organisms. Our method is also applicable to data such as protein-protein and genetic interactions that are categorical in nature. As shown here, m:Explorer is particularly useful in investigating sparse, high-confidence sets of data that may be controversial and not entirely comparable. For instance, we envisage large-scale characterization of human pathways in the context of heterogeneous tumours, utilizing sequence mutations, gene expression and chromatin modification data that are collected in cancer genomics projects.

In our model benchmarks, we demonstrate the advantage of univariate multinomial models in m:Explorer over similar multivariate models (AUC = 0.83 vs AUC = 0.67 for recovering cell cycle TFs). Briefly, the former models treat each TF independently in process gene classification, while the latter models include a non redundant collection of TFs as predictors. However, TF redundancy is an inherent property of robust biological networks that have evolved through gene and genome duplication [68]. In our case, the core cell cycle system involves three pairs of homologous TFs (Swi4 and Swi6; Fkh1 and Fkh2; Ace2 and Swi5) that have strikingly similar TFBS and expression patterns. Due to redundancy, such TFs are not treated as significant predictors in the multivariate setting. This is evident in our simulations: none of the tested multivariate models included both TFs of homologous pairs as significant predictors.

This analysis provides multiple lines of evidence to establish m:Explorer among other methods with similar goals. First, we carried out a highly detailed reconstruction of the known cell cycle regulatory system and proved the validity of our approach through existing knowledge. Second, we repeated the same analysis using eight alternative computational methods and random samples of input data, and provided quantitative proof to the robustness and better performance of our method. Third, we predicted regulators to the enigmatic cellular state of quiescence and validated our top-ranking candidate TFs in follow-up experiments. Nine of twelve tested TFs were confirmed to have consistent and significant G_0 _viability deviations in gene knockout screens, while the remaining three factors showed differences in subsections of our time-course. Thus we proved a high success rate given our relatively simple experimental assays. Besides demonstrating the biological validity of our method, our findings reveal novel, previously unrecognized regulators of quiescence.

### m:Explorer web server and data availability

m:Explorer is available as an R package on our web site [69] and elsewhere. The yeast TF dataset may prove to be a useful resource for the community and is also provided. We have established a web server at [69], allowing online prediction of regulator function using the yeast TF dataset.

## Conclusions

m:Explorer is a generally applicable method for inferring transcription factor function from heterogeneous high-throughput datasets. Our approach outperforms similar state-of-the art tools in recovering regulatory relationships in a well-studied eukaryotic system. Furthermore, the algorithm helps explore uncharacterized regulatory networks and propose valuable hypotheses for detailed assays. Our case study of quiescence G_0 _ and subsequent experimental validations revealed nine novel regulators that enhance or reduce cellular longevity, providing insights to investigators of this cryptic cellular state. In conclusion, our computational and experimental analyses provide strong support to the validity and usefulness of m:Explorer. 

## Materials and methods

### Data processing

The yeast transcription factor dataset of 6253 genes and 285 transcription factors was compiled from gene expression, TF binding and nucleosome positioning data. Perturbation microarrays for 269 regulators were originally produced by Hu *et al*. [1], while our recently reanalyzed dataset [2] was used here for discretized, high-confidence values of up- and down-regulation (moderated t-test, FDR *p *≤ 0.05). Further details on microarray preprocessing are available in the related publication [2]. TF binding site data for 178 TFs were compiled from multiple datasets of ChIP-chip [3], protein-binding microarrays [5] and computational predictions [4,17], using custom filtering and significance cutoffs proposed by the authors. Each promoter of 600 bp was considered to be bound by a TF if at least one binding site occurred in the dataset, and the TFBS was considered nucleosome-depleted (NDTFBS) if nucleosome occupancy [6] at the site was considerably below normalized genome-wide average (t-test, FDR *p *≤ 0.05). Finally, gene expression and TF binding targets for each regulator were integrated and split into eight classes (*up*, *down*, *TFBS*, *NDTFBS*, *up+TFBS*, *down+TFBS*, *up+NDTFBS*, *down+NDTFBS*). All other genes except TF targets were assigned to the baseline class (*not regulated*).

Process-specific gene lists originate from previous high-throughput gene expression experiments. 600 cell cycle specific genes were retrieved from the tiling array experiment by Granovskaia *et al*. [27] and split into six sublists (G1, S, G2, G2/M, M, M/G1) according to authors' instructions. Three classes of diauxic shift genes (early, transient and late expression) originate from the G_0 _time series [35], and genes specific to quiescent and non-quiescent cell cultures were first mapped in the analysis by Aragon *et al*. [36].

### Computational methods

m:Explorer is based on univariate multinomial regression and implements the functionality of the R NNET package [70] for model fitting. We use a list of process-specific genes as categorical model response, and TF target genes as predictors. Briefly, m:Explorer compares two models: the null intercept-only model classifies process gene through their frequency, and the alternative univariate model additionally incorporates TF regulatory targets as predictors. We apply the log likelihood ratio test with null and alternative models to decide if TF target genes are significantly informative of process-related genes. Detailed description of the model is available in Additional file 1.

Yeast cell cycle TFs were predicted from a single structured gene list and directly ranked according to log p-values from m:Explorer. G_0 _TFs were predicted in two independent m:Explorer runs using genes from two datasets. TF p-values from LR tests were log-transformed, scaled to unit range and summed across the two runs to create unbiased composite scores for final ranking. Unit-scaled positive regression coefficients were used to assess the relative phase specificity of cell cycle TFs, since these indicate over-represented regulatory targets in contrast to baseline genes. Relative contribution of regulatory evidence was computed in a similar way.

Linear regression was used to assess the significance of mutant strain viability deviations from control and wildtype strains. With viability as model response *v*, three types of variance were included as model predictors for assessing each mutant/time-point combination across all related replicas, as the alternative model H_1 _: *v ~ i *+ *c *+ *b *+ *m*. The above reflect global variance *i*, variance of negative controls *c*, variance between two batches of independent time-courses *b*, and additional variance of the tested strain *m*. Significance of viability deviation was assessed with a LR test, similarly to the m:Explorer algorithm. Specifically, the null model comprised only global variance, negative control variance and batch variance as H_0 _: *v *~ *i *+ *c *+ *b*, and null and alternative models were compared using the chi-square distribution. Resulting p-values were corrected for multiple testing with FDR.

Fisher's exact tests were used in multiple cases to evaluate the correlation of two binary variables. In the case of TF target genes and cell cycle genes, we applied the Fisher's test to assess whether the proportion of TF-regulated genes was statistically unexpected in the set of cell cycle genes. The Fisher's probability of observing a particular configuration in a two-way contingency table is computed as

$$P(g_{CT},g_{Ct},g_{cT},g_{ct})=\frac{\left(\begin{array}{c} \begin{array}{c} g_{CT}+g_{Ct} \end{array}\\ g_{CT} \end{array}\right)\left(\begin{array}{c} \begin{array}{c} g_{cT}+g_{ct} \end{array}\\ g_{cT} \end{array}\right)}{\left(\begin{array}{c} \begin{array}{c} n \end{array}\\ g_{CT}+g_{cT} \end{array}\right)},$$

where *g *denotes the number of genes in a particular set, *C *indicates cell cycle genes, *T *indicates TF targets, *c *shows genes unrelated to cell cycle, *t *shows genes not regulated by the particular TF, and *n *= *g_CT _*+*g_Ct _*+*g_cT _*+*g_ct _*reflects the number of all yeast genes. As Fisher's test does not support large contingency tables of multi-level variables, different types of TF regulatory targets were treated as the first category and non-regulated genes were assigned to second category, and cell cycle phase-specific genes were similarly merged into a bivariate discrete variable. A similar analysis was carried out to compare the overlap between diauxic shift genes and quiescence genes, using the set of all yeast genes as statistical background.

Gene Ontology (GO) and pathway enrichment analysis for G_0 _TFs was carried out with with g:Profiler software [57]. We defined two ranked gene lists: G_0 _genes [36] that were differentially expressed in WT+ TF knockout strains (Mga2, Cst6, Sds3, Spt10, Bas1), and G_0 _genes that were differentially expressed in viability-deficient TF strains (Swi3, Sin3, Snf2, Spt20, Tup1, Haa1), according to TF knockout microarrays [2]. The gene lists were ordered according to statistical significance in TF knockout data [2], computed as products of p-values across WT+ and RD strains for every gene. We used the ordered enrichment analysis of g:Profiler to find GO functions and pathways in ranked gene lists and applied statistical filtering to find significant enrichments (FDR *p *≤ 0.05).

The one-tailed hypergeometric tests calculated by g:Profiler assess the significance of observing *k *or more genes of a certain functional category in a list of *n *genes, as

$$P\left(x\geq k\right)=\overset{n}{\underset{x=k}{\sum }}\frac{\left(\begin{array}{c} K\\ x \end{array}\right)\left(\begin{array}{c} N-K\\ n-x \end{array}\right)}{\left(\begin{array}{c} N\\ n \end{array}\right)},$$

given that there are *N *genes in total and *K *of which are part of the functional category. As ordered enrichment analysis assumes that genes with stronger signals are ranked first, it consequently tests different subsets of the top list and returns the portion of top genes with the strongest p-value for a particular functional category [71]. Resulting G_0 _functional categories were grouped into three classes: enriched G_0 _categories associating to WT+ TF targets, categories of viability-deficient TF targets, and categories with statistical enrichment in both groups of targets. Enrichment p-values were corrected for multiple testing with the FDR procedure. To rank the third class of common functional categories, we multiplied corresponding p-values of WT+ target genes and viability-deficient TF target genes. After functional enrichment analysis, redundant categories whose genes formed a subset of some other category were removed. To quantify each GO category and function, we also counted up-regulated and down-regulated G_0 _genes across all related TF strains.

## Experimental procedures

Regulator knockout strains were selected as 12 top-ranking candidates from m:Explorer results. *S. cerevisiae *deletion strains originate from the EUROSCARF deletion collection in the BY4741 strain (MATa his3Δ1 leu2Δ0 met15Δ0 ura3Δ0). Liquid cultures were grown in triplicate at 30°C with aeration in YPD (1% yeast extract, 2% peptone, 2% glucose) for 28 days and subsequently shifted to room temperature without aeration. Viability measurements of the six-week time-course were taken in eight time-points: 7h after colony initiation, 48h after colony initiation, followed by six weekly measurements on days 7, 14, 21, 28, 35 and 42. Two independent batches involved distinct sets of tested strains, while wildtypes and controls were covered in both batches. A shorter, independent time-course covered the first three days of growth and involved viability measurements at 7h, 11h, 24h, 48h, and 72h. Cell density was measured at 600 nm. Colony forming units (CFU/ml) were determined by plating cells on YPD agar and counting colonies after three days of growth at 30°C. Culture viability was determined by dividing CFU/ml with total cell number per milliliter in corresponding culture (OD600 units ×10^7^). Growth on glycerol was determined by streaking strains onto YPG plates (1% yeast extract, 2% peptone, 3% glycerol, 2% agar). Glucose concentration was determined by measuring NADPH production in hexokinase and glucose-6-phosphate dehydrogenase coupled reactions provided by Roche.

## Competing interests

The authors declare that they have no competing interests.

## Authors' contributions

Designed and implemented the method: JR. Compiled and analyzed data: JR. Conceived and designed experiments: JR, AA, JMV, JS, NML. Conducted experiments: AA. Wrote the manuscript: JR. Contributed to writing: AA, JV, JMV, JS, NML. All authors have read and approved the manuscript for publication.

## Supplementary Material

Additional file 1 **Supplementary Online Material**. Additional file 1 contains Supplementary Methods, Figures s1-s5 and Tables s1-s3. Click here for file

Additional file 2 **Additional file **2**contains normalized colony forming unit measurements for tested ΔTF strains, wildtypes and controls of the six-week quiescence time-course**. Click here for file

Additional file 3 **Additional file **3**contains normalized colony forming unit measurements for tested ΔTF strains, wildtypes and controls of the 72-hour quiescence time-course**. **Abbreviations**. ΔTF - transcription factor knockout strain; AUC - area under curve; CFU - colony forming units; ChIP - chromatin immunoprecipitation; DNA - deoxyribonucleic acid; FDR - false discovery rate; G_0 _- stationary phase, quiescence; G1 - gap-1; G2 - gap-2; GO - gene ontology; KEGG - Kyoto encyclopedia of genes and genomes; LR - likelihood ratio; M - mitosis; MBF - mlu1-box binding factor; NADPH - nicotinamide adenine dinucleotide phosphate; NDTFBS - nucleosome-depleted transcription factor binding site; OD - optical density; PKA - protein kinase A; PKC - protein kinase C; S - synthesis; SAGA - Spt-Ada-Gcn5-acetyltransferase; SBF - SCB binding factor; TF - transcription factor; TFBS - transcription factor binding site; TOR - target of rapamycin; WT+ - super-wildtype; YPD - yeast extract peptone dextrose; YPG - yeast extract peptone glycerol. Click here for file
