# Supplementary material for: m:Explorer: multinomial regression models reveal positive and negative regulators of longevity in yeast quiescence
Source: Genome Biol. 2012 Jun 21;13(6):R55. doi: 10.1186/gb-2012-13-6-r55 (PMC3446321; doi:10.1186/gb-2012-13-6-r55)
Supplement: Additional file 1 — Supplementary Online Material. Additional file 1 contains Supplementary Methods, Figures s1-s5 and Tables s1-s3. [file gb-2012-13-6-r55-S1.PDF]

SUPPLEMENTARY ONLINE MATERIAL
m:Explorer — multinomial regression models reveal positive and negative regulators of longevity in yeast quiescence

Jüri Reimand*[1,2], Anu Aun[3], Jaak Vilo[2], Juan M Vaquerizas[1], Juhan Sedman[3], Nicholas M. Luscombe*[1,4]

[1]EMBL-European Bioinformatics Institute, Wellcome Trust Genome Campus, Cambridge CB10 1SD, UK
[2]University of Tartu, Institute of Computer Science, Liivi 2, Tartu 50409, Estonia
[3]University of Tartu, Institute of Molecular and Cell Biology, Riia 23, Tartu 51010, Estonia
[4]EMBL-Heidelberg Gene Expression Unit, Meyerhofstrasse 1, Heidelberg D-69117, Germany

Email: Jüri Reimand, *- juri.reimand@utoronto.ca; currently at: Terrence Donnelly Centre for Cellular and Biomolecular Research, University of Toronto, 160 College Street, Toronto, Ontario M5S 3E1, Canada; Anu Aun - anu.aun@ut.ee; Jaak Vilo - vilo@ut.ee; Juan M. Vaquerizas - jvaquerizas@ebi.ac.uk; Juhan Sedman - jsedman@ebc.ee; Nicholas M. Luscombe - luscombe@ebi.ac.uk;

*Corresponding author

Abstract

We developed m:Explorer for identifying process-specific transcription factors (TFs) from multiple genome-wide sources, including transcriptome, DNA-binding and chromatin data. m:Explorer robustly outperforms similar techniques in finding cell cycle TFs in *Saccharomyces cerevisiae*. We predicted and experimentally tested regulators of quiescence (G_0), a model of ageing, over a six-week time-course. We validated nine of top-12 predictions as novel G_0 TFs, including Δ*mga2*, Δ*cst6*, Δ*bas1* with higher viability and G_0-essential TFs Tup1, Swi3. Pathway analysis associates longevity to reduced growth, reprogrammed metabolism and cell wall remodeling. m:Explorer (http://biit.cs.ut.ee/mexplorer/) is instrumental in interrogating eukaryotic regulatory systems using heterogeneous data.

Multinomial logistic regression in m:Explorer

Let the dataset comprise $N = 6253$ genes that are characterized by $K = 285$ predictor variables X_k corresponding to transcription factor (TF) target sets, and one response variable Y corresponding to process-specific gene classification. Let $x_{i,k}$ denote the value of i-th gene with respect to TF k, and y_i the process classification of i-th gene. Let $y_{i,c} = 1$ if y_i is of class c and $y_{i,c} = 0$ otherwise. Both predictor and response variables are represented as multi-class categorical variables, and non-informative classes (gene not related to process, gene not target of TF) are represented as categories of highest ranking.

Multinomial regression is a generalization of linear regression to fit predictor variables with a multi-class ($C \geq 2$) categorical response variable Y, as

$$g(y_i) = \beta_0 + \sum_{k=1}^{K} \beta_k x_{i,k}.$$

The regression coefficients β_k and the intercept β_0 quantify the relationship between K predictors and the response variable. Coefficients inferred using an optimization procedure over all N genes in the dataset, such that agreement between coefficients, predictor values and response values is greatest.

The categorical response values y_i are mapped to numerical space using logistic transformation, such that one class C is selected as baseline, and log odds of all other classes $c \in 1 \ldots C-1$ are computed with respect to baseline, as

$$g(y_i) = \log \frac{p_{i,c}}{p_{i,C}} = \log \frac{p_{i,c}}{1 - \sum_{c=1}^{C-1} p_{i,c}} = \beta_{0,c} + \sum_{k=1}^{K} \beta_{k,c} x_{i,k},$$

where $i \in 1 \ldots N$ reflects genes in the dataset, $c \in 1 \ldots C-1$ corresponds to informative classes in dataset and C is the baseline class, $k \in 1 \ldots K$ represents predictor variables (TF target sets), and $p_{i,c}$ is the probability of value y_i being of class c. Sets of regression coefficients $\beta_{1\ldots k,c}$ as well as intercepts $\beta_{0,c}$ corresponding to $C-1$ distinct response classes are fitted in a joint model. The intercept terms reflect variance in data not covered by other predictors. Specifically, the probability $p_{i,c}$ of gene i belonging to response class c can be expressed through an exponential expression of the predictor variables, as

$$p_{i,c} = \frac{\exp(\beta_{0,c} + \sum_{k=1}^{K} \beta_{k,c} x_{i,k})}{1 + \sum_{c=1}^{C-1} \exp(\beta_{0,c} + \sum_{k=1}^{K} \beta_{k,c} x_{i,k})}$$

for all informative classes $c \in 1 \ldots C-1$ and

$$p_{i,C} = \frac{1}{1 + \sum_{c=1}^{C-1} \exp(\beta_{0,c} + \sum_{k=1}^{K} \beta_{k,c} x_{i,k})}$$

for the baseline class. In the intercept-only model, $p_{i,c}$ corresponds to the fraction of genes in class c.

Model fitting, as implemented in the `nnet` R package of the MASS library [1], involves the inference of regression coefficients β that maximize the agreement between process classification of genes and TF target sets. The agreement is quantified as the likelihood of coefficients given predictor and response variables (the data), such that predicted probabilities of genes are matched to their actual response classes, using the multinomial distribution,

$$L(\beta|Y) = \prod_{i=1}^{N} \frac{(\sum_{c=1}^{C} y_{i,c})!}{\prod_{c=1}^{C} y_{i,c}!} \prod_{c=1}^{C} p_{i,c}^{y_{i,c}} \propto \prod_{i=1}^{N}\prod_{c=1}^{C} p_{i,c}^{y_{i,c}} =$$

$$= \prod_{i=1}^{N}\prod_{c=1}^{C-1} p_{i,c}^{y_{i,c}} p_{i,C}^{y_{i,C}-\sum_{c=1}^{C-1} y_{i,c}} = \prod_{i=1}^{N}\prod_{c=1}^{C-1} (\frac{p_{i,c}}{p_{i,C}})^{y_{i,c}} p_{i,C}^{y_{i,C}},$$

where the term involving the fraction of factorials does not depend on probabilities $p_{i,c}$ and can be omitted from optimization. We can express the probabilities as the combination of predictor variables and convert to the more efficient log likelihood, as

$$l(\beta|Y,X) = \sum_{i=1}^{N}\sum_{c=1}^{C-1} (y_{i,c}(\beta_0 + \sum_{k=1}^{K} \beta_{k,c} x_{i,k})) - y_{i,C} \log(1 + \sum_{c=1}^{C-1} \exp(\beta_{0,c} + \sum_{k=1}^{K} \beta_{k,c} x_{i,k})).$$

Model fitting involves identification of coefficients $\hat{\beta}$ that maximize the likelihood, as

$$\hat{\beta} = \arg\max_{\beta} l(\beta|Y,X),$$

where optimization is carried out with the Broyden–Fletcher–Goldfarb–Shanno (BFGS) method that equates to zero the first partial derivatives of the likelihood function with respect to regression coefficients, as

$$\frac{\partial l(\beta)}{\partial \beta_{k,c}} = \sum_{i=1}^{N} y_{i,c} x_{i,k} - \frac{y_{i,C}}{1 + \sum_{c=1}^{C-1} \exp(\beta_{0,c} + \sum_{k=1}^{K} \beta_{k,c} x_{i,k})} \times \frac{\partial}{\partial \beta_{k,c}} (1 + \sum_{c=1}^{C-1} \exp(\beta_{0,c} + \sum_{k=1}^{K} \beta_{k,c} x_{i,k})) =$$

$$= \sum_{i=1}^{N} y_{i,c} x_{i,k} - y_{i,C} p_{i_c} x_{i,k}.$$

Since there are K coefficients for predictor variables plus an intercept coefficient, and all classes except baseline involve a set of coefficients, $(C-1) \times K$ partial derivatives are involved in the optimization.

m:Explorer employs multinomial regression to compare two hypotheses. The null hypothesis states that a given process-specific classification of genes is determined by the uniform distribution, expressed as the intercept-only multinomial regression model

$$\mathbf{H_0} : g(Y) = \beta_0.$$

The alternative hypothesis states that the process classification is determined by the target sets of a given TF X_k, expressed as the univariate regression model

$$\mathbf{H_1} : g(Y) = \beta_0 + \beta_k X_k.$$

If the predictor variable X_k is categorical and involves $t \geq 2$ classes, it is mapped to mutually exclusive indicator variables $T_{k,1}, \ldots, T_{k,t-1}$ such that the last class is omitted, as

$$g(Y) \sim \beta_0 + \beta_{k,1} T_{k,1} + \cdots + \beta_{k,t-1} T_{k,t-1}.$$

The maximum likelihood estimates $\hat{l}$ of null and alternative models are compared to determine whether TF target genes are significantly informative of process-specific gene classification. The comparison is carried out with the standard log likelihood ratio test that assumes the asymptotic chi-square distribution of log likelihood statistics, given the difference of degrees of freedom ν of the two models, as

$$P(\mathbf{H_0}) = P_{\chi_2}(-2(\hat{l}(\mathbf{H_0}) - \hat{l}(\mathbf{H_1})), \nu_1 - \nu_0).$$

The null hypothesis is rejected if considering TF target genes in process classification provides a significant improvement in model fit. The degrees of freedom of the two models are $\nu_0 = (C-1)$ and $\nu_1 = (C-1)(T-1)$, where C is the number of response classes (process sub-categories) and T the number of predictor classes (different types of TF targets).

For a given process-specific list of genes, m:Explorer analysis involves independent significance testing for all 285 TF target sets. We discard TFs where all regression coefficients of the alternative model are negative and indicate depletion of TF targets. P-values from log-likelihood ratio tests are corrected with the Benjamini-Yekutieli false discovery rate and filtered for statistical significance (cutoff FDR $p = 0.05$).

Comparison with alternative methods

In our comparisons, eight alternative methods were used to recover cell cycle regulators from different combinations of discretized and numeric data, since no method allows exact replication of our analysis.

Multivariate multinomial logistic regression is very similar to the univariate approach of m:Explorer except that multiple TFs are simultaneously used as predictors of process genes. For this benchmark, we were able to use our TF dataset and process genes analogously to m:Explorer. The key difference between the two approaches was the selection of optimal TFs as predictors in the multivariate case, carried out by standard iterative forward-selection procedure implemented in the MASS R package [1]. Briefly, forward selection starts with an intercept-only model and attempts to add predictors from a pool. At each step, the predictor with the greatest improvement in model fit is added to the model, and the procedure terminates when no further predictors are available or the fit ceases to improve. The Akaike Information Criterion $A = 2\nu - 2l$ determines fit, where l is the maximized log likelihood and ν corresponds to model degrees of

freedom. Backward selection from a fully fitted model was not applicable due to over-fitting of the starting point ($l = 0$). The final list of process-specific TFs was extracted from the model with log likelihood ratio tests after the search procedure had converged. In each test, null hypothesis comprised the final model excluding a certain TF, and alternative hypothesis comprised the final model itself. Statistically significant p-values from log likelihood ratio tests served as final scores for TFs ($p \leq 0.05$).

Fisher's exact test is a standard statistical test used for assessing biases in binary contingency tables. In our case, we applied the test to evaluate the association between single TF target genes and cell cycle genes, using our discretized TF dataset and process-specific genes. Since the standard test applies to two-class tables, we merged all informative classes of TF targets (TFBS, ΔTF) into one class, leaving remaining genes in the non-informative baseline class. Process-specific genes were processed similarly. We constructed association tables through two binary vectors of all genes, indicating whether these belonged to TF targets and process genes respectively. All resulting p-values were corrected for multiple testing with the Benjamini-Yekutieli procedure (FDR $p \leq 0.05$) and subsequently used as final TF scores.

Decision trees have been used in the GeneClass algorithm to select discretized microarray and TF binding site profiles that serve as good classifiers of a list of process-related genes [2]. We executed the original implementation of GeneClass algorithm using default parameters without cross validation. Since this method only allows a single list of process genes, we disregarded cell cycle phase information and studied the composite list of 600 cell cycle genes. We also disregarded chromatin state information in TF binding sites. As the unsupervised version of this algorithm trains independent classifiers for gene expression and binding sites, we split our dataset into two separate matrices. GeneClass does not allow direct integration of TF perturbation data and corresponding binding targets. Our first matrix described target genes affected in TF knockouts, and the second matrix mapped target genes of TF binding. Correspondingly, GeneClass returned classifier rankings for TF knockouts and binding sites separately. We assumed that biologically relevant TFs are informative in expression-based classification as well as TFBS classification, and most important TFs are ranked as first classifiers. Therefore the final scores for TFs were computed as sums of inverted ranks of TFs from the two classifiers.

Linear regression models of Transfactivity of the REDUCE package [3] are used to associate TF binding in gene promoter regions with expression values of downstream genes. It provides methods for univariate and multivariate linear regression models. The method uses DNA sequence of gene promoters and position-specific binding motifs as predictors of TF binding affinity, while model response comprises expression values of downstream genes. Although the computational technique of regression models is relatively similar to

m:Explorer, REDUCE requires a substantially different set of data as input. For model predictors, we used the TF binding matrices and promoter sequences provided in the REDUCE package, and the numeric cell cycle gene expression matrix from the study by Granovskaia *et al.* [4] for model response. The tool provided no means to incorporate TF knockout microarrays and chromatin state measurements in TF binding sites. Transfactivity computes a significance p-value for every binding TF and time-point in the cell cycle time-course. To create final scores, we removed insignificant p-values ($p \geq 0.05$) and computed log10 sums of significant TF p-values across all time-points.

Mutual information has been used in the ARACNE algorithm to reconstruct transcriptional networks from numeric gene expression data [5]. Briefly, the algorithm performs data discretization, estimates information theoretic similarity between all gene pairs and finally uses an error model to distinguish statistically significant association from random noise and indirect associations. We applied the adaptive partitioning setting in the ARACNE software with the default significance cutoff ($p = 10^{-7}$) and parameters as provided by ARACNE authors. We performed the comparison in two steps. First, we reconstructed the transcription regulatory network with log fold change values from TF knockout microarrays [6], using the ARACNE option to restrict the set of regulators to our collection of TFs. Second, we applied the Fisher's exact test to associate cell cycle genes with TF targets in the network. We used the composite list of cell cycle genes, since Fisher's test does not support integration of phase-specific lists. The latter step is similar to the MARINA method [7] proposed the authors of ARACNE. This pipeline does not provide direct means to integrate expression data with both TF binding data and gene functional annotations, so the TFBS dataset was excluded from the analysis. Log10 p-values from Fisher's tests were used as final TF scores.

Kolmogorov-Smirnov (KS) test is a standard non-parametric test that has been used specifically to reconstruct the regulatory system of yeast cell cycle [8]. Briefly, the authors used two types of tests to detect TFs with cell cycle activities. First, binding targets of cell cycle TFs were assumed to be significantly differently expressed in contrast to non-targets in at least one cell cycle phase. Second, the binding targets of cell cycle TFs were assumed to show differences in expression across different cell cycle phases, i.e. at least one pair of phases was expected to show significantly altered gene expression of TF-bound target genes. The authors used the KS statistic to evaluate gene expression in all cell cycle phases for the first type of test, and in all pairs of phases for the second type of test. We reproduced the original study with two important differences: first, we introduced the recent cell cycle gene expression time-course [4], and second, we used our high-confidence collection of TFBS compiled from multiple sources [9–11]. We discarded TFs that failed in one or both tests of differential expression ($p \geq 0.05$), as did the original authors, and used the log sum

of most significant p-values from the two types of tests for creating final TF scores.

Biclustering in the SAMBA algorithm has been used for characterizing transcriptional networks and inferring gene function [12]. SAMBA analysis involves organization of molecular data in a matrix of genes in rows and regulators in columns, biclustering of the matrix into two-dimensional clusters, and inference of regulatory relationships between regulators and target genes based on their cluster co-occurrences. We used our dataset of TF targets (TF knockout microarrays, TF binding data, nucleosome occupancy) with cell cycle genes defined by Granovskaia *et al.* [4]. SAMBA was downloaded as part of the Expander software package, and used with default settings (except for final optimization step, see below). Three sources of evidence were extracted from our TF dataset: a) gene expression data from TF knockouts, b) TF binding sites (TFBS) with no nucleosome depletion, c) TFBS with nucleosome depletion. Each evidence source for each TF was presented to SAMBA as a separate property (matrix column), and TFBS and nucleosome-depleted TFBS were represented as discrete data points (1 for TFBS in a gene promoter, 0 otherwise). Gene expression data were also analyzed as discrete values (1 for up-regulation, 0 for no significant change, -1 for down-regulation). Real-valued gene expression log fold changes were used for biclustering in a separate analysis, however these resulted in poorer performance of SAMBA. To integrate cell cycle processes into SAMBA analysis, we identified bi-clusters with significantly over-represented genes of cell cycle phases using Fisher's exact test. To score TFs in relation to cell cycle groups, we computed log-sums of enrichment p-values for all TF properties in respective biclusters (TFBS, TF knockout microarray, TFBS with nucleosome depletion). Prior to log-summing, p-values were corrected and filtered as shown in Expander (Bonferroni multiple testing correction, $p \leq 0.05$). In a separate analysis, we integrated individual cell cycle processes as matrix columns and counted co-occurring regulators for TF scoring, however this approach resulted in poorer performance of SAMBA. Finally, we carried out SAMBA analysis with six sets of parameters predefined in Explorer software, and selected the parameters (valsp_2ap) that provided the best performance in recovering cell cycle TFs.

References

1. Venables WN, Ripley BD: *Modern Applied Statistics with S*. New York, USA: Springer, 4th edition 2003.
2. Middendorf M, Kundaje A, Wiggins C, Freund Y, Leslie C: **Predicting genetic regulatory response using classification**. *Bioinformatics* 2004, **20 Suppl 1**:i232–240.
3. Bussemaker HJ, Li H, Siggia ED: **Regulatory element detection using correlation with expression**. *Nat. Genet.* 2001, **27**:167–171.
4. Granovskaia MV, Jensen LJ, Ritchie ME, Toedling J, Ning Y, Bork P, Huber W, Steinmetz LM: **High-resolution transcription atlas of the mitotic cell cycle in budding yeast**. *Genome Biol.* 2010, **11**:R24.
5. Margolin AA, Nemenman I, Basso K, Wiggins C, Stolovitzky G, Dalla Favera R, Califano A: **ARACNE: an algorithm for the reconstruction of gene regulatory networks in a mammalian cellular context**. *BMC Bioinformatics* 2006, **7 Suppl 1**:S7.
6. Reimand J, Vaquerizas JM, Todd AE, Vilo J, Luscombe NM: **Comprehensive reanalysis of transcription factor knockout expression data in Saccharomyces cerevisiae reveals many new targets**. *Nucleic Acids Res.* 2010, **38**:4768–4777.
7. Lefebvre C, Rajbhandari P, Alvarez MJ, Bandaru P, Lim WK, Sato M, Wang K, Sumazin P, Kustagi M, Bisikirska BC, Basso K, Beltrao P, Krogan N, Gautier J, Dalla-Favera R, Califano A: **A human B-cell interactome identifies MYB and FOXM1 as master regulators of proliferation in germinal centers**. *Mol. Syst. Biol.* 2010, **6**:377.
8. Tsai HK, Lu HH, Li WH: **Statistical methods for identifying yeast cell cycle transcription factors**. *Proc. Natl. Acad. Sci. U.S.A.* 2005, **102**:13532–13537.
9. Harbison CT, Gordon DB, Lee TI, Rinaldi NJ, Macisaac KD, Danford TW, Hannett NM, Tagne JB, Reynolds DB, Yoo J, Jennings EG, Zeitlinger J, Pokholok DK, Kellis M, Rolfe PA, Takusagawa KT, Lander ES, Gifford DK, Fraenkel E, Young RA: **Transcriptional regulatory code of a eukaryotic genome**. *Nature* 2004, **431**:99–104.
10. MacIsaac KD, Wang T, Gordon DB, Gifford DK, Stormo GD, Fraenkel E: **An improved map of conserved regulatory sites for Saccharomyces cerevisiae**. *BMC Bioinformatics* 2006, **7**:113.
11. Zhu C, Byers KJ, McCord RP, Shi Z, Berger MF, Newburger DE, Saulrieta K, Smith Z, Shah MV, Radhakrishnan M, Philippakis AA, Hu Y, De Masi F, Pacek M, Rolfs A, Murthy T, Labaer J, Bulyk ML: **High-resolution DNA-binding specificity analysis of yeast transcription factors**. *Genome Res.* 2009, **19**:556–566.
12. Tanay A, Sharan R, Kupiec M, Shamir R: **Revealing modularity and organization in the yeast molecular network by integrated analysis of highly heterogeneous genomewide data**. *Proc. Natl. Acad. Sci. U.S.A.* 2004, **101**(9):2981–2986.
13. Steinmetz LM, Scharfe C, Deutschbauer AM, Mokranjac D, Herman ZS, Jones T, Chu AM, Giaever G, Prokisch H, Oefner PJ, Davis RW: **Systematic screen for human disease genes in yeast**. *Nat. Genet.* 2002, **31**:400–404.
14. Dimmer KS, Fritz S, Fuchs F, Messerschmitt M, Weinbach N, Neupert W, Westermann B: **Genetic basis of mitochondrial function and morphology in Saccharomyces cerevisiae**. *Mol. Biol. Cell* 2002, **13**:847–853.

ORF	Name	Description
YMR043W	MCM1	Transcription factor involved in cell-type-specific transcription and pheromone response; plays a central role in the formation of both repressor and activator complexes
YDL056W	MBP1	Transcription factor involved in regulation of cell cycle progression from G1 to S phase, forms a complex with Swi6p that binds to MluI cell cycle box regulatory element in promoters of DNA synthesis genes
YOR372C	NDD1	Transcriptional activator essential for nuclear division; localized to the nucleus; essential component of the mechanism that activates the expression of a set of late-S-phase-specific genes
YER111C	SWI4	DNA binding component of the SBF complex (Swi4p-Swi6p), a transcriptional activator that in concert with MBF (Mbp1-Swi6p) regulates late G1-specific transcription of targets including cyclins and genes required for DNA synthesis and repair
YDR146C	SWI5	Transcription factor that activates transcription of genes expressed at the M/G1 phase boundary and in G1 phase; localization to the nucleus occurs during G1 and appears to be regulated by phosphorylation by Cdc28p kinase
YLR182W	SWI6	Transcription cofactor, forms complexes with Swi4p and Mbp1p to regulate transcription at the G1/S transition; involved in meiotic gene expression; cell wall stress induces phosphorylation by Mpk1p, which regulates Swi6p localization
YIL131C	FKH1	Forkhead family transcription factor with a minor role in the expression of G2/M phase genes; negatively regulates transcriptional elongation; positive role in chromatin silencing at HML and HMR; regulates donor preference during switching
YNL068C	FKH2	Forkhead family transcription factor with a major role in the expression of G2/M phase genes; positively regulates transcriptional elongation; negative role in chromatin silencing at HML and HMR; substrate of the Cdc28p/Clb5p kinase
YLR131C	ACE2	Transcription factor that activates expression of early G1-specific genes, localizes to daughter cell nuclei after cytokinesis and delays G1 progression in daughters, localization is regulated by phosphorylation; potential Cdc28p substrate

Table s1: 9 core cell cycle TFs.

ORF	Name	Description
YML027W	YOX1	Homeodomain-containing transcriptional repressor, binds to Mcm1p and to early cell cycle boxes (ECBs) in the promoters of cell cycle-regulated genes expressed in M/G1 phase; expression is cell cycle-regulated; potential Cdc28p substrate
YNL309W	STB1	Protein with a role in regulation of MBF-specific transcription at Start, phosphorylated by Cln-Cdc28p kinases in vitro; unphosphorylated form binds Swi6p and binding is required for Stb1p function; expression is cell-cycle regulated
YDL106C	PHO2	Homeobox transcription factor; regulatory targets include genes involved in phosphate metabolism; binds cooperatively with Pho4p to the PHO5 promoter; phosphorylation of Pho2p facilitates interaction with Pho4p
YOR344C	TYE7	Serine-rich protein that contains a basic-helix-loop-helix (bHLH) DNA binding motif; binds E-boxes of glycolytic genes and contributes to their activation; may function as a transcriptional activator in Ty1-mediated gene expression
YDR451C	YHP1	One of two homeobox transcriptional repressors (see also Yox1p), that bind to Mcm1p and to early cell cycle box (ECB) elements of cell cycle regulated genes, thereby restricting ECB-mediated transcription to the M/G1 interval
YGR044C	RME1	Zinc finger protein involved in control of meiosis; prevents meiosis by repressing IME1 expression and promotes mitosis by activating CLN2 expression; directly repressed by a1-alpha2 regulator; mediates cell type control of sporulation
YCR065W	HCM1	Forkhead transcription factor that drives S-phase specific expression of genes involved in chromosome segregation, spindle dynamics, and budding; suppressor of calmodulin mutants with specific SPB assembly defects; telomere maintenance role
YKL185W	ASH1	Zinc-finger inhibitor of HO transcription; mRNA is localized and translated in the distal tip of anaphase cells, resulting in accumulation of Ash1p in daughter cell nuclei and inhibition of HO expression; potential Cdc28p substrate
YHR206W	SKN7	Nuclear response regulator and transcription factor, part of a branched two-component signalling system; required for optimal induction of heat-shock genes in response to oxidative stress; involved in osmoregulation

Table s2: 9 secondary cell cycle TFs.

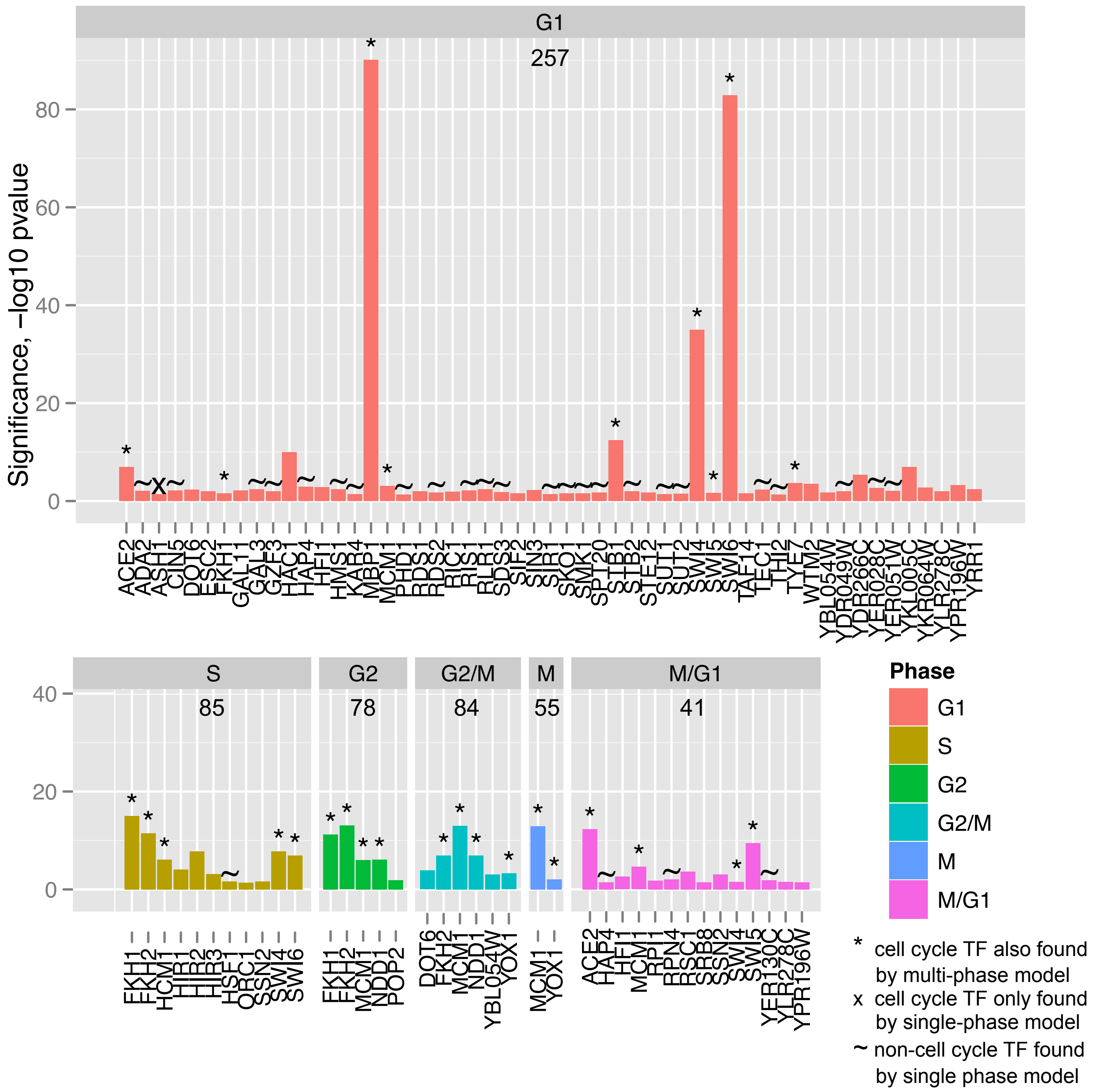

Figure s1: m:Explorer TF predictions for cell cycle genes, performed independently for 4 phases and 2 checkpoints with m:Explorer using logistic regression. Numbers in plot headers show phase-associated genes from the gene expression time-course [4]. Symbols above bars show predicted TFs in comparison to the multi-phase model in **Figure 3A**. Single-phase cell cycle models appear to predict many more false positive candidate TFs, denoted by ∼ symbols.

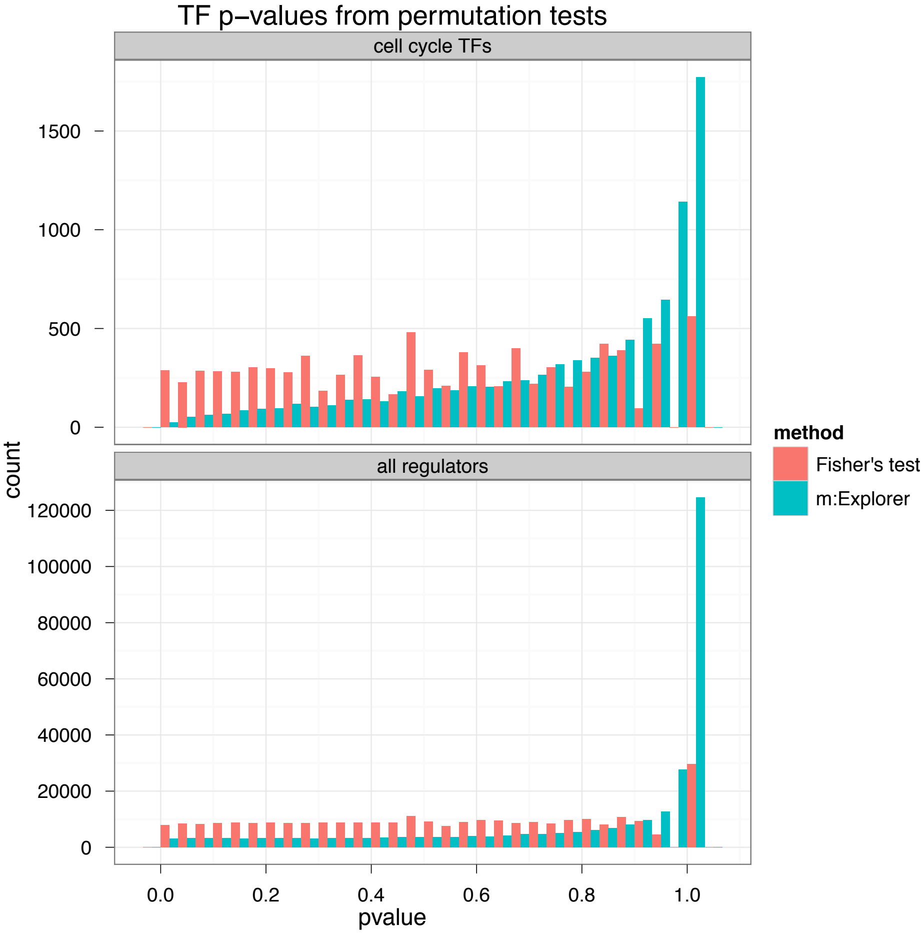

Figure s2: m:Explorer and Fisher's exact test were compared in permutation tests, by shuffling TF targets and finding associations to cell cycle genes for 1000 times for each TF. Distribution of resulting non-corrected p-values is plotted for 9 cell cycle TFs (top) and all TFs (bottom), for Fisher's test (red) and m:Explorer (green). Greater occurrence of high p-values in m:Explorer predictions indicates that our method is less prone to false positive discovery.

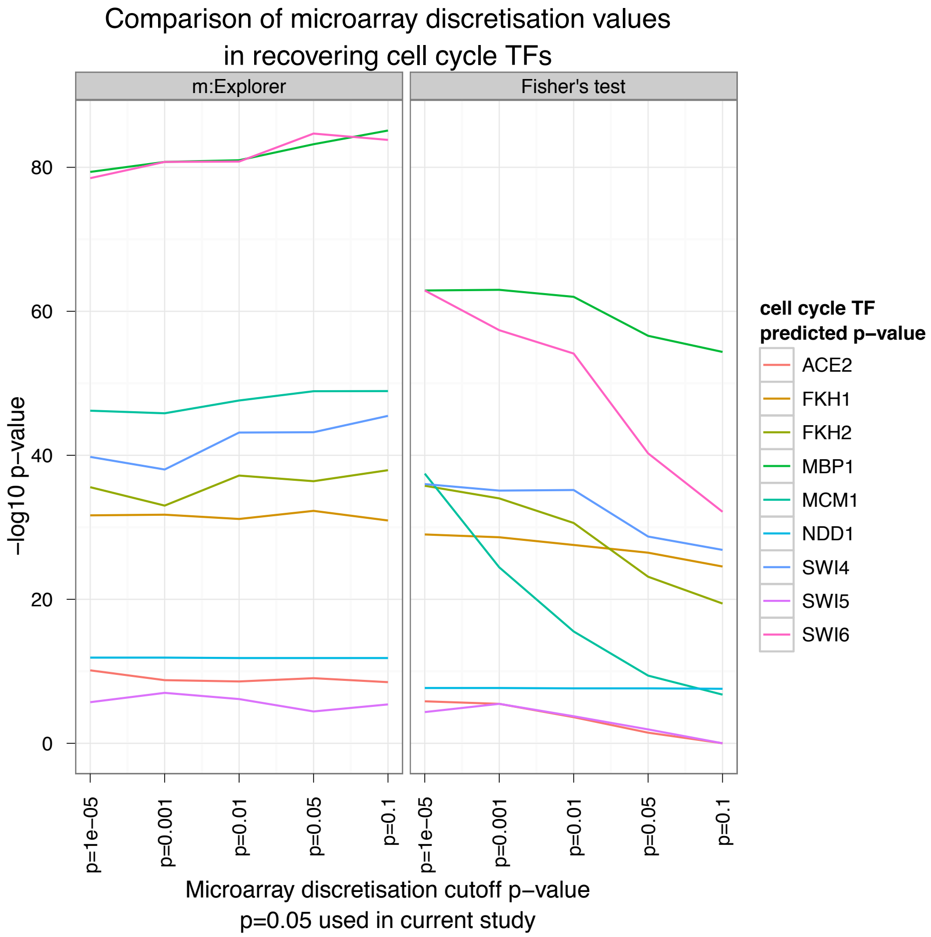

Figure s3: m:Explorer and Fisher's exact test were studied in the context of discretization of TF deletion microarrays [6]. We re-compiled the TF target datasets using four alternative cutoff values and compared the performance of the two methods in recovering cell cycle TFs. In conclusion, m:Explorer in robust to microarray discretization, as successfully recovers all core TFs and appears to show increased sensitivity with more liberal cutoff values. In contrast, Fisher's test results become less significant as the microarray cutoff is reduced. Specifically, it fails to recover Swi5 and Ace2 at cutoff $p = 0.1$.

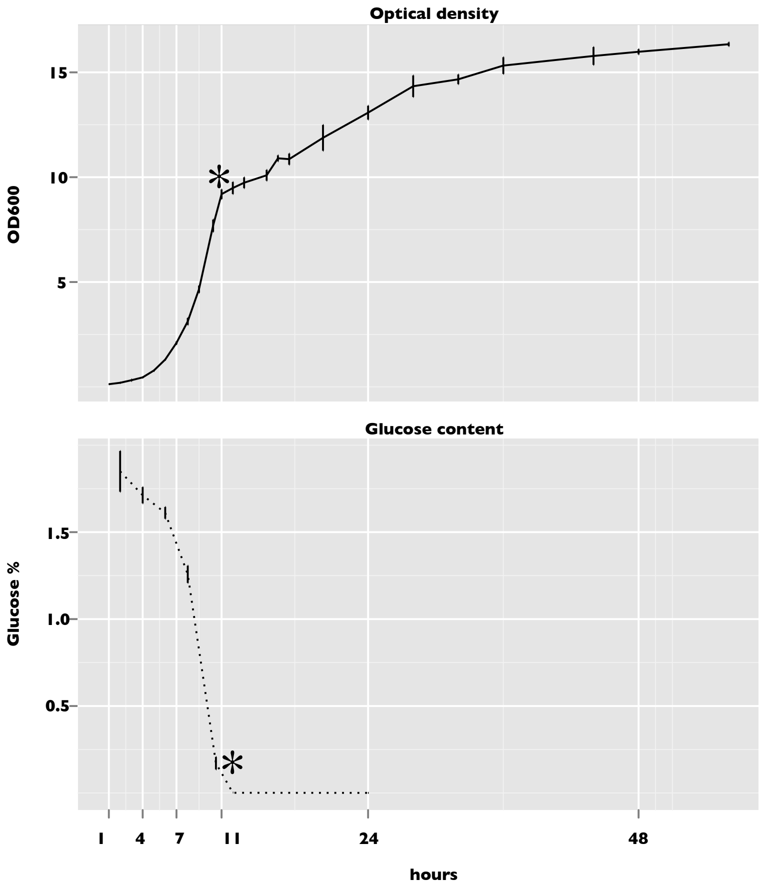

Figure s4: Temporal occurrence of diauxic shift was determined through measurements of culture density (OD600, top plot) and glycose concentration (bottom plot). Asterisk at 11 hours indicates simultaneous depletion of glycose and switch from exponential to logarithmic growth.

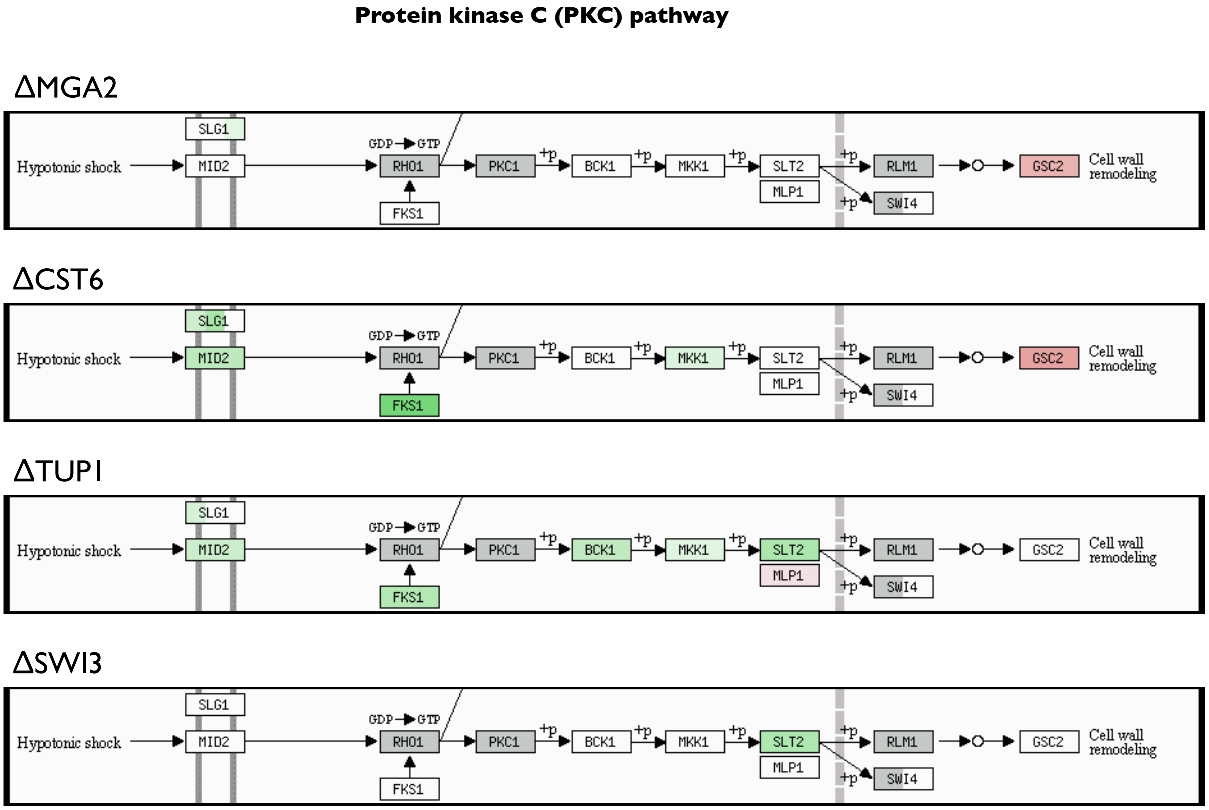

Figure s5: Protein kinase C (PKC) pathway and cell wall remodelling shows differential regulation in WT+ strains ($\Delta mga2$, $\Delta cst6$) and viability-deficient strains ($\Delta tup1$, $\Delta swi3$).

Strain	Class	Growth YPG	Respiratory growth Steinmetz [13], 2002	Respiratory growth Dimmer [14], 2002
	wildtype	+		
Δ*mip1*	positive control	-	decreased	absent
Δ*ard1*	positive control	+	decreased	
Δ*gal3*	negative control	+		
Δ*pdr3*	negative control	+		
Δ*mga2*	super-wildtype	+		
Δ*cst6*	super-wildtype	+	decreased	absent
Δ*sds3*	super-wildtype	+		
Δ*bas1*	super-wildtype	+		
Δ*spt10*	super-wildtype	+		
Δ*tup1*	viability deficient	-		absent
Δ*swi3*	viability deficient	-	decreased	absent
Δ*sin3*	viability deficient	+		
Δ*haa1*	viability deficient	+		
Δ*snf2*	no viability deviation in late G_0	-*	decreased	absent
Δ*spt20*	no viability deviation in late G_0	-		absent
Δ*snf11*	no viability deviation in late G_0	+		

Table s3: Respiratory ability of selected ΔTF strains was assessed by streaking cultures to glycerol medium (YPG). Evidence from previous large-scale screens is shown in the two rightmost columns. Contrary to previous evidence, Δ*cst6* successfully grows in YPG medium, whereas our Δ*cst6* strains produce WT+ phenotypes in G_0. Asterisk in Δ*snf2* denotes observed micro-colonies.
